# Identification and Functional Demonstration of miRNAs in the Fungus *Cryptococcus neoformans*

**DOI:** 10.1371/journal.pone.0052734

**Published:** 2012-12-26

**Authors:** Nan Jiang, Yaping Yang, Guilhem Janbon, Jiao Pan, Xudong Zhu

**Affiliations:** 1 Department of Microbiology, College of Life Sciences, Nankai University, Tianjin, China; 2 Tianjin Institute of Industrial Biotechnology, Chinese Academy of Sciences, Tianjin, China; 3 Department of Molecular Mycology, Institute Pasteur, Paris, France; New Jersey Medical School, University of Medicine and Dentistry of New Jersey, United States of America

## Abstract

microRNAs (miRNAs), endogenous posttranscriptional repressors by base-pairing of their cognate mRNAs in plants and animals, have mostly been thought lost in the kingdom of fungi. Here, we report the identification of miRNAs from the fungus *Cryptococcus neoformans*. With bioinformatics and Northern blotting approaches, we found that these miRNAs and their hairpin precursors were present in this fungus. The size of miR1 and miR2 is 22 nt and 18 nt, respectively. The precursors are about ∼70 nt in length that is close to mammalian pre-miRNAs. Characteristic features of miRNAs are also found in miR1/2. We demonstrated that the identified miRNAs, miR1 and miR2, caused transgene silencing via the canonical RNAi pathway. Bioinformantics analysis helps to reveal a number of identical sequences of the miR1/2 in transposable elements (TEs) and pseudogenes, prompting us to think that fungal miRNAs might be involved in the regulation of the activity of transposons and the expression of pseudogenes. This study identified functional miRNAs in *C. neoformans*, and sheds light on the diversity and evolutionary origin of eukaryotic miRNAs.

## Introduction

Small non-coding RNAs (sRNAs) ranging from 20 to 30 nucleotides (nt) in length are widely found in the major phyla of eukaryotes [1]–[3]. Many of them have been shown to function as regulatory agents in RNA interference (RNAi) networks [4], [5]. By precursor structure, biogenesis origin, and mode of action, sRNAs are generally categorized into three classes, *i.e.* microRNAs (miRNAs), small interfering RNAs (siRNAs), and piwi-interacting RNAs (piRNAs) [4], [6], [7]. The class miRNA is featured by its endogenous origin and its flexibility in base-pairing to cause degradation of target mRNA [2], [8], [9]. miRNAs are derived from larger stem-looped hairpin precursors that are transcribed from miRNA genes and are processed by Dicer or Dicer-like proteins [7], [8], [10]. Usually, they act as posttranscriptional repressors of the target gene to cause silencing via binding partially or perfectly to the complementary sequences located in the 3′untranslated regions (3′-UTRs) or the other part of target mRNAs [2], [3], [11], [12].

Gene silencing by RNAi is also conserved many fungi. Diverse small RNAs (sRNAs) and RNAi pathways have been described from a number of fungi [13], [14]. As a matter of fact, most fungal sRNAs are siRNAs, for instance, in the yeast *Schizosaccharomyces pombe* [15], budding yeast *Saccharomyces castellii*, *Candida albicans* and *Cryptococcus neoformans* [16]–[19], as well as in the filamentous fungus *Neurospora crassa* [13], [14]. In *N. crassa*, miRNA-like sRNAs (milRNAs) were lately reported [14]. Twenty five milRNAs were predicted based on non-coding precursor RNA genes that contain inverted RNA sequences to form hairpin structure by bioinformatics analysis. Some milRNAs are formed from different pathways other than the conventional pathways that generate miRNAs in animals and plants [14]. However, a typical miRNA in fungi has been largely elusive by far.

*C. neoformans* is an opportunistic human pathogen for cryptococcosis in immuno-compromised patients, *e.g.* AIDS-afflicted people [20]. The fungus possesses the core components for RNAi process, including Argonaute proteins (encoded by *AGO1* and *AGO2*, mainly the *AGO1*), Dicers (*DCR1* and *DCR2*, mainly *DCR2*) and RNA-dependent RNA polymerase (*RDP1*) [19], [21], [22]. The RNAi machinery in *C. neoformans* is functional as transgenic dsRNA has been used to knock down the target genes [18]. A sex-induced silencing of transgenes (multiple copies in tandem) was observed via RNAi during the sexual stage in this fungus [19]. Deep sequencing of siRNAs in *C. neoformans* revealed a portion of siRNAs were derived from putative repetitive transposon-like sequences, and the biogenesis of these siRNAs depended on the function of RNAi machinery [19]. Here, we report the identification of microRNA in *C. neoformans*. After sequencing approximately 200 of small RNAs of ∼20–25 nt, two of them, designated as miR1 and miR2, were proven to have the characteristic features of miRNAs. miR1/2 was likely derived from 70-nt RNA precursors that were revealed by bioinformatics analysis and by Northern blotting. When miR1/2 was fused to reporter genes, *URA5* and *CLC1*, at the 3′ untranslated region, *URA5* and *CLC1* were silenced at the posttranscriptional level, which was RNAi machinery-dependent. Both miR1 and miR2 were mapped to the transposable elements (TEs) and pseudogenes.

## Results

### Cloning and analyses of small RNAs in C. neoformans

**Figure 1:**
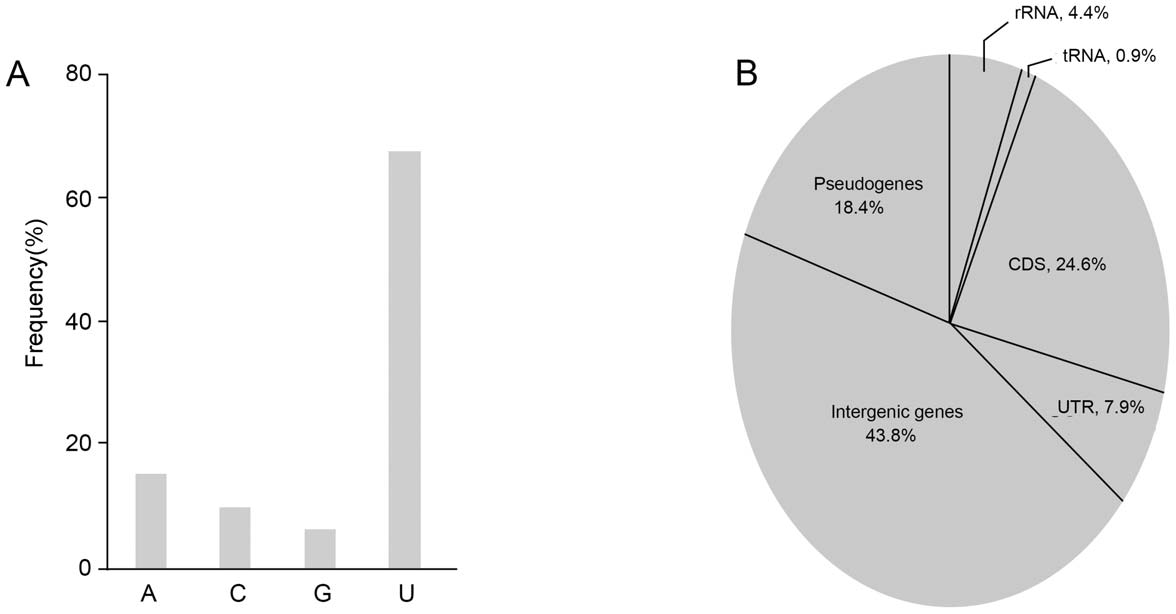
Statistic diagram of small RNAs in *C. neoformans* B4500. (A) Frequency of the nucleotides at the 5′ end of the sequenced small RNAs. (B) Genomic distribution of sRNAs in *C. neoformans*.

To comprehensively search for miRNAs, a library for small RNAs (20–25 nt) was constructed for a two-day-old culture of *C. neoformans*, containing approximately 20000 clones. Two hundred clones were randomly selected for sequencing. The size of the inserts ranged from 14 to 25 nt. The majority of them (67.5% of the total) had a preference of U at the 5′ end (Figure 1A), a characteristic hallmark of small RNAs from animals, plants and other fungi [2], [14], [19]. To find out the distribution pattern of these sRNAs in the genome, we made blasting alignment against GenBank databases. As shown in Figure 1B, a small fraction (5.3%) of the cloned sRNAs perfectly matched the sequences of rRNAs (4.4%) and tRNAs (0.9%). And 32.5% of them hit transcriptional products, covering both coding sequences (CDS, 24.6%) and 3′-untranslated region (UTR, 7.9%). The rest (62.2%) of the sRNAs matched pseudogenes and intergenic regions. This distribution pattern is remarkably close to the data reported by other laboratories from different organisms [14], [19]. That sRNAs match to multiple loci indicates the diverse origination of sRNAs at this range of size. Obviously, the function of these sRNAs remains a challenging issue to address for researchers.

### Identification of miRNA in C. neoformans

**Figure 2:**
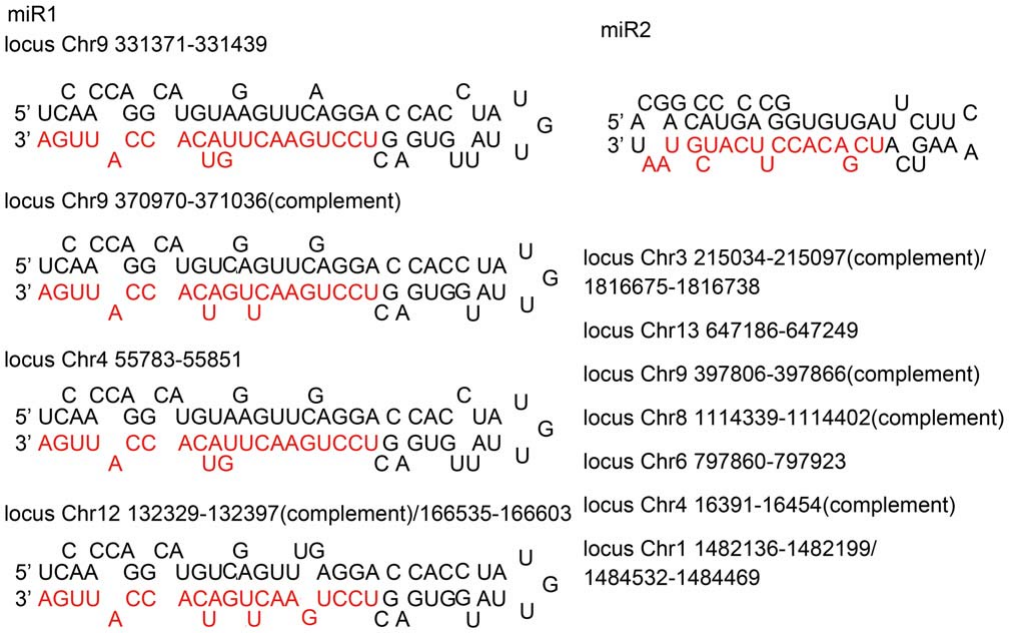
Structure of the predicted pre-miRNAs of miR1 and miR2. The sequences of miR1 and miR2 are highlighted by red letters.

To screen for cryptococcal miRNA in the sequenced sRNA library, the following criteria were taken into account. First, with bioinformatics tools such as software Vmir (http://books.elsevier.com/companions/ 978012379179), we examined the loci of sRNAs in the genome for precursor transcripts with hairpin structures that harbor a sRNA on the stem [8]. Secondly, these sRNAs that had more than three homologous loci in the genome were chosen except for those from the rRNA and tRNA loci. And most importantly, the presence of the sRNAs could be detected by Northern blotting. By these standards, two miRNA candidates, miR1 (22 nt) and miR2 (18 nt), were acquired. Both miR1 and miR2 begins with a U at the 5′ end and are located on the arm of putative hairpin-form RNA precursors (Figure 1 and 2) [14]. The miRs are distributed on several highly complementary loci in the genome. For instance, five loci have identical sequence to miR1, while nine loci perfectly match miR2. Most of these loci encode transposable elements (TEs) and pseudogenes (Table 1). A few others are protein-encoding or intergenic regions. The possible precursors (pre-miRNA) were also found by computation with Vmir and other software that predicts hairpin structures (Figure 2) (carried out by commercial service).

**Table 1:** Chromosomal distributions of miR1 and miR2.

Name/Length	Chr.	Loci	Identities (%)	Coding
miR1/22 nt5′-TCCTGAACTTGATCACCATTGA	Chr12	132350–132329	100	Protein AAW 46453.1
		166582–166603	100	Transposase_21 (AAW 46457.1)
	Chr4	55830–55851	100	Protein XM 570676.1
	Chr9	331418–331439	100	Intergenic gene
		370991–370970	100	pseudogene
	Chr1	1365317–1365335	86.4	Intergenic gene
		2111331–2111318	63.6	Protein AAW41297.1
miR2/18 nt5′-TCGACACCTTCATCGTAA	Chr3	215067–215049	100	MULE (AAW42067.1)/pseudogene
		1816706–1816723	100	Protein AAW42575.1
	Chr13	647216–647234	100	Protein AAW46987.1/AAW46986.1
	Chr9	397836–397818	100	MULE (AAW45547.1)
		1139994–1139982	72.2	Intergenic gene
	Chr8	1114372–1114354	100	MULE (AAW44911.1)
	Chr6	797890–797908	100	MULE (AAW44276.1)
		81752–81739	77.8	Protein AAW44202.1
	Chr4	16424–16406	100	MULE (AAW43368.1)/pseudogene
		1656773–1656760	77.8	Chromatin Remodeling Complex (AAW43182.1)
	Chr1	1482167–1482184	100	Protein AAW41058.1
		1484501–1484484	100	MULE (AAW41060.1)
		788546–788534	72.2	Intergenic gene

**Figure 3:**
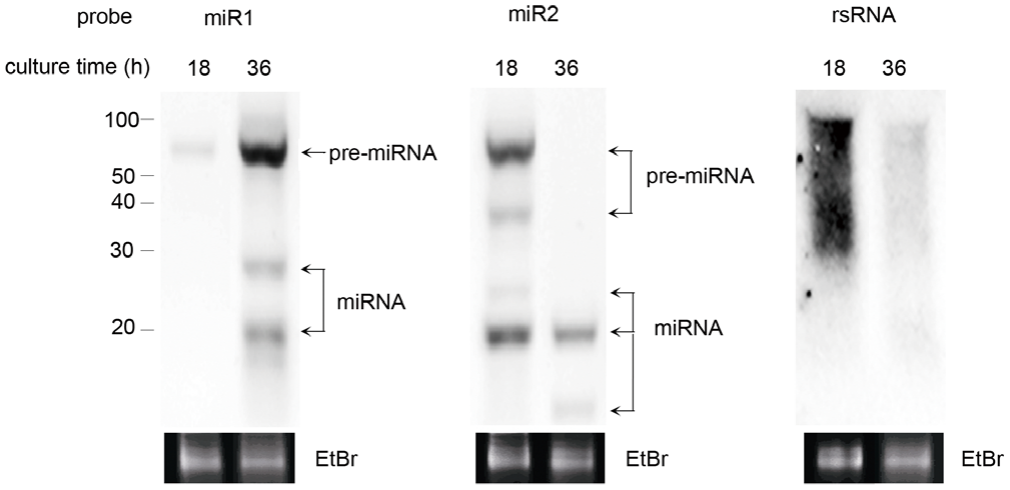
Detection of miRNAs by Northern blotting. Positive bands for miR1 and miR2 (the left two panels) and their precursors were detected. On the right, rsRNA, a randomly picked small RNA only formed a smear band signal. Probes were the synthesized DNA sequence corresponding to miR1, miR2 or rsRNA. Total RNA was prepared from cultures collected at 18 hr and 36 hr as indicated. EtBr stands for ethium bromide-stained gel showing the 22-nt miRNA bands. Approximately 10 µg of total RNA was equally loaded. The size of the RNA markers is shown on left.

Distinct from other sRNAs, miRNAs are processed from larger precursors of ∼70-nt in animals, and from 47 to 698 nt in plants [23], [24]. It is critical to demonstrate the existence of miR1/2 and their precursors in *C. neoformans*. Therefore, we carried out Northern blotting to detect miR1/miR2 and their precursors (Figure 3). Probes for the blots were miR1, miR2, respectively. A third sRNA randomly picked in the library (rsRNA) served as control (see Materials and Methods). RNA samples were prepared from yeast cells grown for 18 and 36 hours. As anticipated, miR1, miR2 and their precursors were detected in the blots (two left panels, Figure 3). Pre-miR1 was approximately 70 nt in size which was close to the length of mammalian miRNAs [14]. A couple bands between the 20-nt and 70-nt bands were seen, suggesting that intermediate steps may be required for the maturation of miR1 (left panel, Figure 3). Interestingly, expression of miR1 and miR2 seemed to be related to culture time. At 18 hr, miR1 was hardly detectable and its precursor pre-miR1 started to be transcribed. At 36 hr, both pre-miR1 and miR1 were seen at a high level. For miRNA2, a higher expression level was observed at 18 hr than at 36 hr (the central panel, Figure 3). A similar precursor was confirmed for miR2 at ∼70 nt. Two more intermediate bands were also seen for miR2. It is noteworthy that only smeared signal was obtained in the blot for the control sRNA that did not has interfering effect (Right panel, Figure 3).

### Demonstration of URA5-miRs silencing

**Figure 4:**
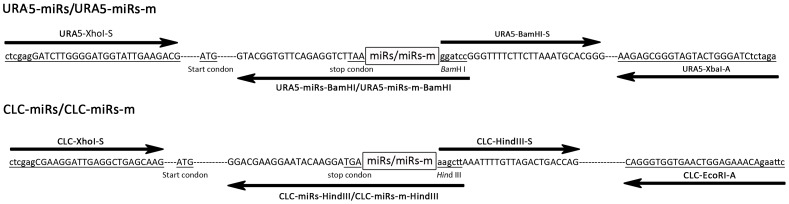
Schematic diagram of the construction of reporters in silencing assay. The upper panel shows the construction of URA5-miRs or URA5-miR-ms. Two pair of primers, URA5-XhoI-S/URA5-miRs-BamHI and URA5-BamHI-S/URA5-XbaI-A, were used to PCR amplify the ORF and the terminator regions of *URA5*, respectively. Among the primers, URA5-miRs-BamHI harbored the sequence of miRs or miR-ms through primer design. The two PCR fragments were then ligated after digested by *BamH* I. Similarly, the construction of *CLC-miR1/2* and *CLC-miR1-m/miR2-m* was made (the bottom part). The position of miRs or miR-ms is indicated by the boxes. Restriction sites are in small letters. Arrows mark the position and direction of the primers. Start codons and stop codons of *URA5* and *CLC1* are underlined. For detailed description, see the section of Materials and Methods.

Since miRNAs regulate target mRNAs through complementary base-pairing [25], we constructed a fusion gene, *URA5-miR1* and *URA5-miR2*, as reporter to examine the interfering activity of miR1 and miR2 (see the section Materials and Methods, Figure 4). miR1 or miR2 was inserted by a PCR method at the 3′ UTR of *URA5* which encodes an enzyme in the biosynthesis of uracil [26] (Figure 4). In principle, silencing *Ura5* would yield auxotrophic phenotype for uracil and resistance to the toxin 5-fluoro-orotic acid (5-FOA). Plasmids carrying respectively *URA5-miR1* and *URA5-miR2*, were introduced into a recipient strain B4500FOA (*ura5*-) by electroporation. For control, half of the sequence (50% of the nucleotides) of miR1 and miR2 were randomly mutated to generate miR1-m and miR2-m that were likewise fused to *URA5* (Materials and Methods). All transformants were selected on appropriate plates containing 100 µg/ml hygromycin (the plasmids contained hygromycin B resistant marker).

**Figure 5:**
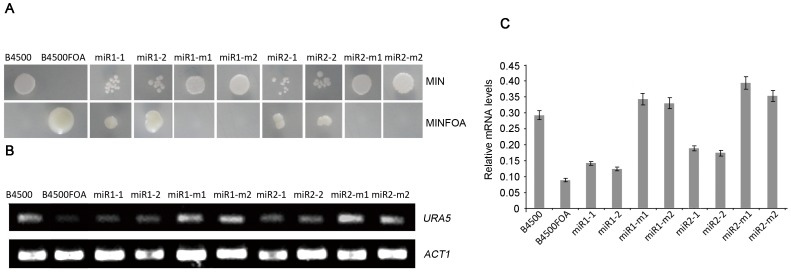
Silencing of the reporter gene *URA5-miRs*. (A) The upper panels show a slower growth rate of the transformants of *URA5-miR1* (two transformants was picked in each case, namely, miR1-1 and miR1-2), and *URA5-miR2* (miR2-1 and miR2-2), than the wild type B4500, and the transformants of miR1-m (*i.e.* miR1-m1 and m2) and miR2-m, on MIN agar supplemented with 100 µg/ml hygromycin B, no hygromycin for B4500 and B4500FOA. The negative control B4500FOA (*ura5*) did not grow on MIN. On MINFOA (the bottom panels), the wild type strains and transformants of miR1-m and miR2-m were killed by FOA. Transformants containing miR1 and miR2, as well as *ura5* ^−^ strain B4500FOA grew properly. MIN: minimal medium, and MINFOA: minimal medium with 5-FOA and 50 mg/l uracil. For selection of transformants, 100 µg/ml hygromycin B was added to MIN or MINFOA when needed. (B) Reverse transcription-PCR confirmed the decreased mRNA level of *URA5* in *URA5-miR* transformants. *URA5* mRNA levels in the wild type and in the transformants of miR-ms were close to each other. In each assay, two transformants were picked for double examination. (C) A semi-quantification of *URA5* mRNA to *ACT1* mRNA in the strains in (B).

Transformants were tested for uracil prototrophy on selective medium MIN (minimal medium without uracil). Two transformants of *URA5-miR1* (miR1-1 and miR1-2, Figure 5A) and *URA5-miR2* (miR2-1 and miR2-2, Figure 5A) grew significantly slower than the wild type B4500 (and the negative control B4500FOA failed to grow). In contrast, transformants of miR1-m and miR2-m (miR1-m1/miR1-m2, and miR2-m1/miR2-m2, respectively) exhibited a similar growth rate to the wild type B4500 (the upper panels of Figure 5A). In a concomitance, transformants of *URA5-miR1* and *URA5-miR2* survived in the presence of 5-FOA, whereas transformants carrying mutated miR1/miR2 (miR1-m1/miR1-m2 and miR2-m1/miR2-m2 in Figure 5A), failed to grow, as well as the wild type B4500 (the bottom panels in Figure 5A). These results clearly demonstrated that miR1 and miR2 had a silencing effect on the expression of *URA5*. When miR1 and miR2 were mutated, the silencing did not occur to the reporter *URA5*.

The relative transcriptional level of *URA5* was determined by semi-quantitative reverse transcription-PCR (with the software Quantity One, BioRad, CA, USA). The result was shown in Figure 5B. The level of *URA5* mRNA in the transformants of *URA5-miR1* and *URA5-miR2* was significantly lower than that in the wild type B4500 and the non-silencing controls (miR1-m1/miR1-m2, or miR2-m1/miR2-m2) (Figure 5C). When the relative abundance of *URA5* mRNA in each sample was calculated against the internal standard *ACT1* mRNA, the ratio of the transcripts in the miR1-1, 2 and miR2-1, 2 was 0.13±0.013 and 0.18±0.009, significantly lower than the value for the wild type B4500 (0.29±0.01), and the controls, miR1-m1/2 (0.34±0.0098) and miR2-m1/2 (0.37±0.028), confirming that *URA5* expression was knocked down by miRs.

Still more, quantitative real time PCR (qRT-PCR) was adopted for further verification of *URA5-miRs* silencing in the transformants. Ratio of *URA5* to the internal standard *ACT1* mRNA was calculated in each strain. As shown in Table 2, *URA5* mRNA decreased by 6 and 4 fold respectively in the miR1 and miR2 transformants compared to the wild type. In a striking contrast, in transformants of miR1-m1, 2 and miR1-m1,2, expression level of *URA5* was close to the wild type, which was significantly higher than in the knock-down transformants. Putting together, by phenotypic assays, reverse transcription PCR and qRT-PCR, we have confirmed that miRNAs, miR1 and miR2, have interfering activity in *C. neoformans*.

**Table 2:** Expression of *URA5-miRs* determined by qRT-PCR in different transformants.

strains	ΔC_T_	ΔΔC_T_	Normalized
	(Avg.*URA5*C_T_-Avg.*ACT1*C_T_)	(Avg.ΔC_T_-Avg.ΔC_T, B4500_)	*URA5* amount relative to B4500 2^−ΔΔC^_T_
B4500	2.42±0.19	0.00±0.19	0.88–1.14
miR1	4.48±0.12	2.07±0.13	0.22–0.26
miR2	4.97±0.05	2.55±0.15	0.15–0.19
miR1-m	2.54±0.17	0.12±0.03	0.90–0.94
miR2-m	2.07±0.14	−0.35±0.10	1.2–1.4

### The silencing of reporter CLC-miRs

Using another gene *CLC1* as reporter, we here present one more example to show gene silencing caused by miR1 and miR2. *CLC1* encodes a chloride channel that is essential for pigment formation in the presence of polyphenolic substrate in *C. neoformans* [27]–[29]. *CLC1* disruption results in albino yeast colonies on nor-epinephrine (NE)-containing agar. The reporter construct *CLC-miRs* were made in a similar way for *URA5-miRs* (Figure 4). The recipient strain was a disruption mutant of *CLC1*, *Δclc1* (melanin-deficiency, see Materials and Methods) [28].

**Figure 6:**
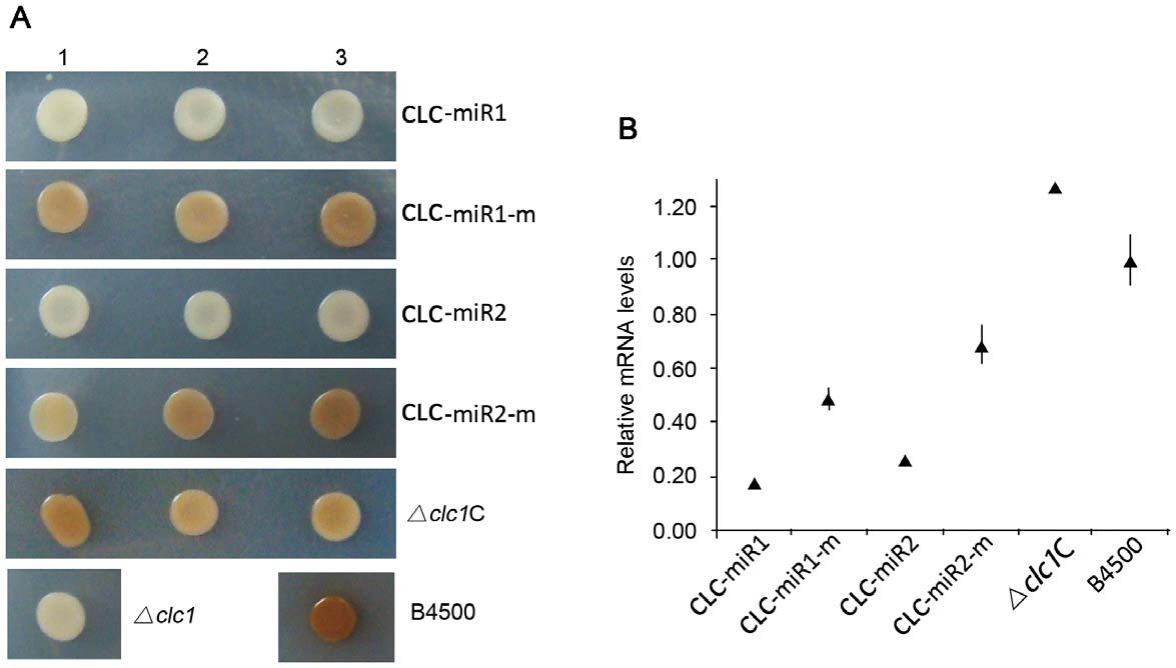
Silencing of the reporter *CLC-miRs*. A similar silencing assay was carried out for the reporter *CLC-miRs*. (A) Melanin-deficient phenotype of the transformants of *CLC-miRs* (#1 to #3) was observed as expected, suggesting knockdown expression of *CLC1*. When miRs were mutated, *CLC-miR-ms* restored melanin production (Second and forth rows from the top). Cells were placed on low-glucose (0.1%) Asn agar with NE and 100 µg/ml hygromycin except for the wild types. (B) Decreased mRNA level of *CLC1* by miRs silencing determined by reverse transcription-PCR for the strains in (A). Relative abundance of *CLC1* mRNA verse actin-encoding gene *ACT1* mRNA was considered. PCR reaction was performed in triplicate.

As anticipated, transformants of *CLC-miRs* (# 1,2 and 3) formed light colonies on NE plates, a phenotype similar to *Δclc1* strain, suggesting a silencing outcome of *CLC*-*miR1/2* in the transformants (the first and the third rows from the top, Figure 6A). The transformants of *CLC1*- miR1-m1 or miR2-m restored to produce pigment on NE plates, a phenotype similar to the wild type B4500 and the complement strain *Δclc1C* (Figure 6A). The ratio of *CLC1* mRNA to *ACT1* mRNA by reverse transcription PCR confirmed the knockdown of *CLC1* expression in the transformant of miR1 and miR2 (Figure 6B). In addition, remarkable down-regulation of *CLC1* mRNA was confirmed by qRT-PCR in transformants of miR1 and miR2 (Table 3), *i.e.* approximately 2.8- and 2.6-fold lower than that in the wild type.

**Table 3:** Expression of *CLC1-miRs* determined by qRT-PCR in different strains.

strains	ΔC_T_	ΔΔCT	Normalized
	(Avg.*CLC*C_T_-Avg.*ACT1*C_T_)	(Avg.ΔCT-Avg.ΔC_T, B4500_)	CLC amount relative to B4500 2^−ΔΔC^_T_
B4500	4.30±0.13	0.00±0.13	0.91–1.09
*ΔCLC1C*	3.95±0.02	−0.35±0.02	1.26–1.29
CLC-miR1	6.81±0.02	2.51±0.02	0.17–0.18
CLC-miR2	6.24±0.07	1.94±0.07	0.25–0.27
CLC-miR1-m	5.34±0.12	1.04±0.12	0.45–0.53
CLC-miR2-m	4.85±0.15	0.55±0.15	0.62–0.76

In summary, through silencing assays for reporter *URA5* and *CLC1*, we demonstrated that miRNA-mediated gene silencing occurs in *C. neoformans*.

### miRNA-mediated gene silencing via canonical RNAi machinery in C. neoformans

**Figure 7:**
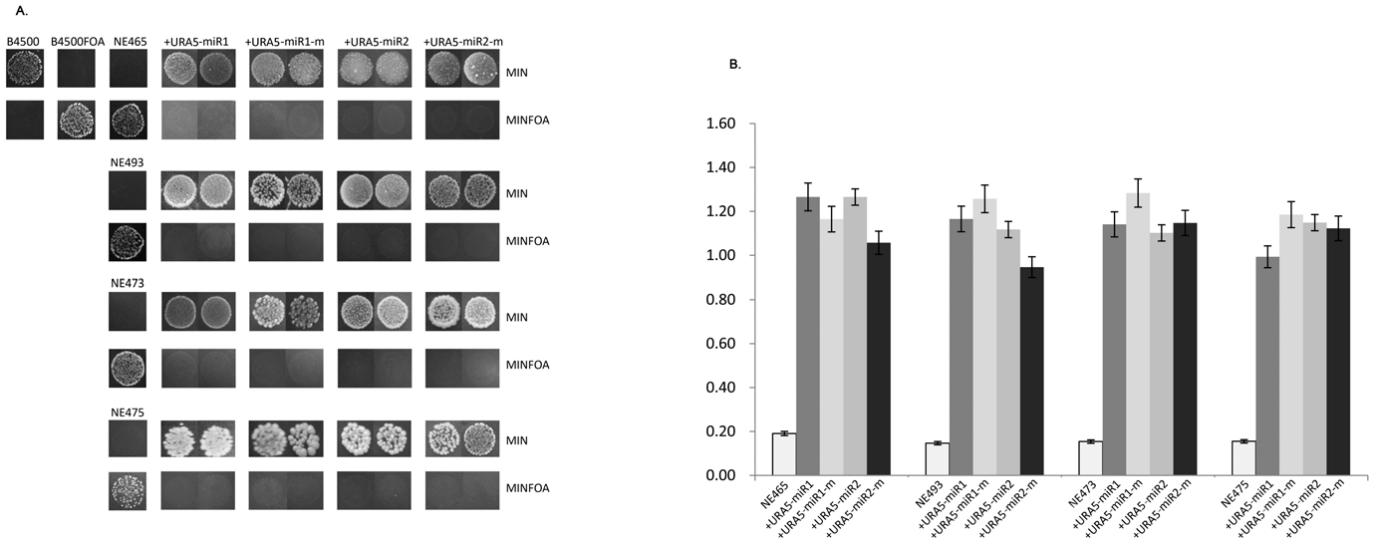
miR-mediated gene silencing requires RNAi machinery in *C. neoformans*. (A) *URA5-miR1/2* restored the growth of B4500FOA (*ura5*) on MIN agar in the RNAi-deficient mutant strains, NE465, NE493, NE473 and NE475, *i.e.* gene silencing of *URA5-miRs* that was observed in Fig. 4 did not occur in these mutants. And these transformants failed to grow on plates containing 5-FOA (the panels of MINFOA agar). MIN or MINFOA agar was supplemented with 100 µg/ml hygromycin B for the selection of all transformants of the reporters. The drug was left out for B4500 and B4500FOA. The transformants of miR1-m and miR2-m, together with the wild type B4500 and B4500FOA, served as control. (B) qRT-PCR confirmation of restored expression of *URA5* in RNAi-deficient mutants, NE465, NE493, NE473 and NE475, which are originally *ura5* defective strains.

We further found that miRNA-mediated gene silencing involves the RNAi machinery in this fungus. The reporter silencing assay was carried out in several mutant strains in which components required for the RNAi have been deleted. These strains include NE465 (*ago1Δ::NAT-STM*, *ura5*), NE493 (*rdp1Δ::NAT-STM ura5*), NE473 (*dcr1Δ::NEO*, *ura5*), and NE475 (*dcr21Δ::HYG-STM*, *ura5*) [30]. Likewise, plasmids carrying *URA5-miR1* and *URA5-miR2*, and their corresponding mutated forms (as control), were introduced into the mutants, separately. *URA5-miR1* and *URA5-miR2* restored the auxotrophic phenotype of all the strains (*ura5^−^*) (Figure 7A), suggesting that gene silencing was abolished in the RNAi-deficient mutants. In contrast, transformants with the mutated form of miR1 and miR2 were able to grow on minimal media (without uracil). All the transformants failed to grow in the presence of 5-FOA, reinforcing that *URA5-miRs* was appropriately expressed in RNAi-defective mutants (Figure 7A). Still more, qRT-PCR ratified that the reporter *URA5-miRs* was properly expressed in the mutants of *ago1*, *rdp1*, and *dcr1*, *dcr2* (Figure 7B). Taking together, the above results suggest that miRNA-mediated gene silencing occurs via RNAi pathway in *C. neoformans*.

## Discussion

In this work, we identified two miRNAs, miR1 (22 nt) and miR2 (18 nt), from the basidiomycetous yeast *C. neoformans*. They carry structural hallmarks of conventional miRNAs defined by studies in other eukaryotic organisms, for instance, a U at the 5′ end of the small RNAs [14]. We found that miR1 and miR2 are putatively derived from stem-loop RNA precursors by bioinformatics approach (Figure 2). Besides, precursor RNAs, together with miR1 and miR2, were detected by Northern blotting (Figure 3). Notably, the pre-miR1 and pre-miR2 are approximately 70 nt in size and is close to the length of mammalian miRNAs [14]. Northern blots also showed that expression of miR1 and miR2 depended on culture time, suggesting that action of cryptococcal miRs is under regulation. We used different approaches to show that miR1 and miR2 have interfering activity of small RNAs. We designated two reporters, *URA5-miRs* and *CLC1-miRs*, with miR1 and miR2 inserted at the 3′ UTR of *URA5* and *CLC1*, accordingly, in the rational that miRNAs cause gene silencing via base-paring of the homologous sequence on the target mRNA [3], [12]. These reporters were indeed silenced when introduced in the fungus (Figure 5 and 6). Molecular data generated by qRT-PCR or reverse transcription PCR made a further confirmation of the dramatic decrease of the reporter genes, *URA5* and *CLC by* miR1 and miR2 (Figure 5, Figure 6 and Figure 7).

Further more, we showed that the silencing caused by miR1 and miR2 was actually realized through RNAi pathway in *C. neoformans*. We took advantage of four available mutant strains NE465 (ago1Δ::NAT-STM, *ura5*), NE493 (rdp1Δ::NAT-STM *ura5*), NE473 (dcr1Δ::NEO, *ura5*), and NE475 (dcr21Δ::HYG-STM, *ura5*), in which RNAi components, *AGO1* for Argonaute, *RDP1* for RNA-dependent RNA polymerase and *DCR1* and *DCR2* for two Dicer proteins, were separately disrupted [30]. As shown in Panel A of Figure 7, disruption of the genes in any of the mutants demonlished *URA5-miRs*-caused gene silencing. As a result, transformants were unable to grow on 5-FOA-containing plates (the *ura5* ^−^ controls grew on 5-FOA). Also, qRT-PCR confirmed that there was no discernable knock-down effect for the reporter gene *URA5-miR1* or *URA5-miR2* in the transformants (Panel B, Figure 7). These results clearly demonstrate that miRs-mediated gene silencing is realized via RNAi machinery in *C. neoformans*.

Distribution of miRNAs across the genome may represent the targets or pre-RNA genes of the miRNAs. Via bioinformatics analysis, identical sequences of miR1 and miR2 are revealed on four and seven chromosomes, respectively (Table 1). Seven homologs of miR1 were found in the genome, five of them share 100% identity. Four of them are putatively located within a hairpin RNA structure (Figure 2). Whereas, thirteen homologous loci of miR2 were located over the genome, nine of them harbor identical miR2. Homologous loci of miR1 are located in pseudogenes, intergenic genes and transposable elements (TEs) (Table 1). Loci of miR2 can be divided into two groups: the long TEs and unknown protein family, including AAW46987.1, AAW41058.1, AAW42575.1 and AAW46986.1. Putative conserved domains of TEs loci were detected in the sequences surrounding both miRs [31]. In the loci of protein AAW 46457.1 that harbors miR1, peptide sequence from residues 245 to 406 shares a identity of 81.9% to the cryptococcal Tnp2-like transposase (Transposase_21) [32]. The ORFs containing miR2, AAW 42067.1, AAW41060.1, AAW45547.1, AAW44276.1 and AAW44911.1, putatively encode FHY3 (Far-red Elongated Hypocotyl 3) as well as MULE (Mutator-like Transposable Element) domains [32]. These TEs may be the target of miRNA-mediated silencing, or likely as the biogenesis source of the miRs. A previous study showed that RNAi pathway in *C. neoformans* serotype D controls the mobility of transposons [31]. In this respect, the biological significance of miRNA-mediated gene silencing may lie on the fact that miR1 and miR2 inhibit transposon activity. Experimental efforts have been made under the way to illustrate the possible targets of the miRs in this fungus. Based on our data, it appears safe to draw a conclusion that miR1 and miR2 at least plays a role in defending the genome integrity by interfering transgene expression in *C. neoformans*.

On the other hand, TEs likely serve as the genomic source for the biogenesis of miRNAs. This is very likely for miR1 in *C. neoformans* as four miR1-containing loci harbor a hairpin RNA structure (Figure 2). A similar case was reported in human, the family of miRNA genes, has-mir-548, are derived from Made1 transposable elements that are short miniature inverted-repeat transposable elements [33], [34]. In plants, miRNAs are also encoded by a number of individual TE insertions [35], [36]. More than 30% of miRNA genes in plants are associated with interspersed repeats [36]. It is believed that the dispersed repetitive nature of TE sequences provides multiple novel pre-miRNA genes as well as serves as target sites throughout the genome [33].

Our data indicate that pseudogenes may play a role in miRNA-associated gene silencing, despite that pseudogenes were thought for a long time as non-functional relics of evolution. A recent study revised this view by showing that transcripts produced from pseudogenes regulate the effects of miRNAs on their targets by competing for miRNA binding. Poliseno *et al.* showed that *PTENP1*, a pseudogene, can derepress their cognate genes, because they retain many of the miRNA binding sites and can compete for the binding of many miRNAs at once [37]. And the pseudogenes were reported as microRNA decoys lately [38]. It is intriguing to believe that cryptococcal pseudogenes may have an intrinsic biological role in miRNA regulatory networks.

It should be noted that miR1 and miR2 share little similarity to known miRNAs in plants and animals by sequence comparison (E-value>0.1), supporting an independent evolution view of miRNAs in fungi [14]. The discovery of functional miRNAs in *C. neoformans* would generate significant effect on gene regulation network in this fungi. And accurate investigation on the function of miR1 and miR2 is also critical for the pathogenesis studies of *C. neoformans*. However, open questions, such as the exact biogenesis of the miRNAs, the elusive targets of miR1 and miR2, and their relationship to pseudogenes, are still challenging.

## Materials and Methods

### Strains and Growth Conditions

*C. neoformans* serotype D strain B4500 (also named as JEC21, ATCC MYA-565) was used as the wild type in this study. B4500FOA is a uracil auxotrophic mutant [39]. *Δclc1* is CLC-type chloride channel mutant derived from B4500 [28]. Both were used as recipient strains for gene silencing assay. Other recipient strains included NE465 (*ago1Δ::NAT-STM*, *ura5*), NE493 (*rdp1Δ::NAT-STM*, *ura5*), NE473 (*dcr1Δ::NEO*, *ura5*), and NE475 (*dcr2Δ::HYG-STM*, *ura5*), in which RNAi components, *AGO1* for Argonaute, *RDP1* for RNA-dependent RNA polymerase and *DCR1* and *DCR2* for two Dicer proteins, were separately disrupted, in that order [30]. The *ΔclcC* strain was a reconstituted strain in which a wild-type copy of *CLC1* was transformed into *Δclc1* strain [28]. YPD medium (2% glucose, 2% bacto-peptone, 1% yeast extract) was used for routine growth of *C. neoformans*. For testing uracil auxotrophy, minimal medium (MIN; 1.7 g yeast nitrogen base without amino acids and ammonium sulfate, 5 g ammomium sulfate, 20 g glucose per 1 liter) were supplemented with 1 g/l 5-fluoroorotic acid (5-FOA) and 50 mg/l uracil for make MINFOA. Low-glucose (0.1%) asparagine salt medium (Asn, 0.1% asparagine, 0.3% KH_2_PO_4_; pH 5.2) was used for melanin production in the presence of the laccase substrate NE (100 mg/liter), at 28°C, for two days [40]. All transformants were selected on YPD or other indicated media such as Asn or MIN containing 100 µg/ml hygromycin (the plasmids contained hygromycin B resistant marker).

### Cloning of small RNAs

Total RNA was isolated from 48-hr culture using traditional guanidinium thiocyanate-phenol protocal. Small RNAs were separated on 15% PAGE urea (7 M) gels with the Small RNA Gel Extraction Kit (TaKaRa Biotechnology, Dalian Co. Ltd, Dalian, China) by following the manufacturer's instruction. A RNA ladder ranging from 20 to 100 was used as the marker. The small RNA band at approximately (22 nt were cut from the PAGE gel and cloned with the Small RNA Cloning Kit (TaKaRa Biotechnology, Dalian Co. Ltd, Dalian, China). Briefly, the purified sRNAs were ligated with the 3′ and 5′ RNA adaptors supplemented with the Kit. cDNA was consequently synthesized and amplified with the supplemented oligo primers. The cDNA were cloned into T-vector pMD20-T supplemented with the Kit to generate sRNA library. The bacterial clones containing sRNAs were randomly picked for sequencing (BGI, Shenzhen, China).

### Identification of miRNAs via bioinformatics analysis

All sequences were searched against the *C. neoforman*s genome in NCBI, as well as SGTC, Stanford Genome Technology Center: *Cryptococcus neoformans* Genome Project (http://sequence-www.stanford.edu/group/C.neoformans/). To determine miRNA candidates, sRNA from tRNA or rRNA were first excluded. Bioinformatics analysis was conducted to predict miRNAs and their location in the genome (carried out by BGI, Shenzhen, China). Structural features of cloned small RNAs was defined by comparison to known plant or mammalian miRNAs. The flanking upstream and downstream regions of miRNA candidates were screened for potential hairpin structure by the software VMir (http://books.elsevier.com/companions/978012379179) (BGI, Shenzhen, China). The final miRNA candidates were further verified by Northern blotting (next part in this section). Targets of the miRNAs were predicted by homology to sequences on chromosomes using bioinformatics tools such as TargetScan and miRanda (finished by BGI, Shenchen, China), and checked by blasting against cryptococcal genome project on GenBank.

### Northern blot analyses

To confirm the existence of miRNA, Northern blotting analysis was conducted. Approximately 10 µg of total RNAs were separated on 15% denaturing PAGE with 7 M urea and transferred onto a Nylon Hybridization Membrane N^+^ (Osmonics, USA) which had been pre-wetted in distilled water. Crosslinking of RNA to the membrane was mediated by carbodimide at 60°C for 70 min [41]. Synthesized DNA probes corresponding to miR1 or miR2 were labeled with γ-[^32^P] ATP using T4 polynucleotide kinase (TaKaRa Biotechnology, China). Hybridization was performed at 37°C in high-efficiency hybrid-buffer (Beijing Biodev-tech Scientific & Technical Co., Ltd. China).

### Reporter constructs and Transformation

Constructs for gene silencing assay were designed as following (Figure 4): a candidate miRNA (miR1 or miR2 in this case) was inserted into the reporter genes, *URA5* or *CLC1*, at their 3′ UTR, to create fusion genes *URA5-miR1/2* or *CLC-miR1/2*. Briefly, for the construction of URA5-miRs fusion gene, two pair of primers, URA5-XhoI-S/URA5-miRs-BamHI and URA5-BamHI-S/URA5-XbaI-A, were used to PCR amplify the ORF and the terminator regions of the wild-type *URA5*, respectively. The primer URA5-miRs-BamHI harbored miR1 or miR2 (Figure 4). The PCR fragments were cut with *Xho* I, *BamH* I and *Xba* I, accordingly and were ligated onto the plasmid pBluescript-Hyg digested with *Xho* I and *Xba* I. The plasmid carries hygromycin B resistance cassette for selection. All the primers used for fragment amplification were collected in Table 4.

**Table 4:** Primers used in this study.

No.	Primers	Sequence
1	CLC–EcoRI-A	GGG TGA ATT CCG AAG GAT TGA GGC TGA GCA AG
2	CLC–XhoI-S	CCT TCT CGA GTG TTT CTC CAG TTC ACC ACC CTG
3	CLC-mi1 -HindIII	CCC GAA GCT TTC CTG AAC TTG ATC ACC ATT GAT CAT CCT TGT ATT CCT TCG TCC
4	CLC-mi1-mut-HindIII	CCC GAA GCT TTC CTG AAT CCA GCT GTT ATT GAT CAT CCT TGT ATT CCT TCG TCC
5	CLC-mi2 -HindIII	CCG CAA GCT TTT ATC GAC ACC TTC ATC GTA ATC ATC CTT GTA TTC CTT CGT CC
6	CLC-mi2-mut-HindIII	CCG CAA GCT TTT ATC GGT GTT CCT GCT GTA ATC ATC CTT GTA TTC CTT CGT CC
7	CLC-HindIII-S	CCC CGA AGC TTA AAT TTT GTT AGA CTG ACC AG
8	URA5-XhoI-S	GGG ACT CGA GGA TCT TGG GGA TGG TAT TGA AGA CG
9	URA5-XbaI-A	CTC GTC TAG AGA TCC CAG TAC TAC CCG CTC TT
10	URA5-mi1-BamHI	GCC CGG ATC CTC CTG AAC TTG ATC ACC ATT GAT TAA GAC CTC TGA ACA CCG TAC
11	URA5-mi1-mut-BamHI	GCC CGG ATC CTC CTG AAT CCA GCT GTT ATT GAT TAA GAC CTC TGA ACA CCG TAC
12	URA5-mi2-BamHI	GCC CGG ATC CTT ATC GAC ACC TTC ATC GTA ATT AAG ACC TCT GAA CAC CGT AC
13	URA5-mi2-mut-BamHI	GCC CGG ATC CTT ATC GGT GTT CCT GCT GTA ATT AAG ACC TCT GAA CAC CGT AC
14	URA5-BamHI-S	TGG GGA TCC GGG TTT TCT TCT TAA ATG CAC GGG
15	qURA5	TAC AGG AGG TTG AGG AAG A(up)
		TTA AGA CCT CTG AAC ACC G(down)
16	qCLC1	AAT GAC TGT GAA TAC GGG CG(up)
		CTT GGT CGG ACA CGA GAA TG(down)
17	qACT	ATG GTA TTG CCG ACC GTA TGC(up)
		TTT CGG TGG ACG ATT GAG GG(down)

As control, miR1 and 2 were mutated to created miR1-m or miR2-m. The sequence of miR1-m was 5′-TCCTGAATCCAGCTGTTTAACT. Nucleotides from 8 to 17 in miR1 were changed. The sequence of miR2-m was 5′-TCGGTGTTCCTGCTGATT. Nucleotides from 4 to 14 were changed. By the same strategy with PCR, miR1-m and miR2-m were fused to *URA5* or *CLC1* in the place of miR1 or miR2 (Figure 4). The construction of the reporters CLC-mi*R1 or 2* and CLC-miR1-m *or miR2-m* was carried out in the same way.

Recipient cells, *e.g.* B4500FOA or *Δclc1* cells were transformed by electroporation as described [42] using 5 µg of linearized DNA (*URA5-miR1/2* or *CLC-miR1/2*) per 3×10^8^ cells. Transformants were first selected on YPD containing 100 µg/ml hygromycin B and purified by streaking. Two or three transformants were picked for phenotype analysis on MIN, MINFOA, or Asn agar supplemented with 100 µg/ml hygromycin in the assays. For verification of the transformed reporters in the transformants, PCR amplification of the reporter genes including the miRs was conducted. The primers, HYG-200:5′- AAGTGCCGATAAACATAACGA and & URA5-XbaI: 5′-CTCGTCTAGAGATCC CAGTACTACCCGCTCTT, were designed to amplify *URA5-miRs* or *URA5-miR-ms*. The resulted 2.7-kb fragment was subject to sequencing. The pair of primers, T7 & CLC-EcoRI:5′-GTAATACGACTCACTATAGGGC/5′-GGGTGAATTCCGAAGGATTGA GGCTGAGCAAG, were used to amplify the *CLC1* reporter. A 4.2-kb fragment was generated and sequenced.

### qRT-PCR assay

Quantitative real-time PCR (qRT-PCR) was carried out with Eppendorf Mastercycler Realplex Detection System. Briefly, total RNA was purified from two-day fungal culture as previously described [43], [44] and treated with DNase I (60–80 U/µl, TaKaRa). Approximately 1 µg DNase-treated RNA was reverse transcribed with M-MLV (RNase H^−^) (TaKaRa, Dalian, China) using oligo(dT)_15_ primer. cDNAs (∼50 ng) was added to 45 µl qRT-PCR reaction mixture containing 25 µl FastStart Universal SYBR Green Master (Roche China, Shanghai, China) and 300 nM of each primers. Each reaction was conducted in triplicate and non-reverse-transcribed RNA samples were used as control. Gene-specific primers (Table 4) were designed using Primer Premier 5 (Premier Biosoft, California, USA), and each pair was validated by cDNA template titration to ensure similar amplification kinetics and a single melting point of quantitative PCR products. To calculate the expression levels of the target gene, relative quantification 2^−ΔΔCT^ method was employed [32]. The housekeeping gene for beta-actin, encoded by *ACT1*, was used as an internal control in this study.
